# Shedding light on decahedral nanoparticle catalysts

**DOI:** 10.1107/S2052252519005621

**Published:** 2019-04-30

**Authors:** Adam Slabon

**Affiliations:** a Department of Materials and Environmental Chemistry, Stockholm University, Svante Arrhenius väg 16 C, 106 91 Stockholm, Sweden

**Keywords:** multiply twinned structures, decahedral nanoparticles, bimetallic, heterogeneous catalysis, electrocatalysts, CO_2_ reduction

## Abstract

Control over the chemical composition and atomic ordering in bimetallic nanoparticles has driven the recent rapid progress in electrocatalysis. Liang & Yu [*IUCrJ* (2019), **6**, 447–453] elucidate the structural differences between single metal and intermetallic multiply twinned decahedral nanoparticles on the example of AuCu particles and correlate the catalytic properties with their structure at the atomic level.

Nanoparticles are of high importance for several technical applications, including energy storage, optoelectronics and heterogeneous catalysis. For example, gold and copper nanoparticles are active catalysts for the electrochemical reduction of carbon dioxide (Hori *et al.*, 2002 ▸). Consequently, nanoparticles of gold or copper could be applied in electrolyzers that are empowered by external photovoltaic modules to reduce carbon dioxide to carbon-based fuels in an environment-friendly process. In addition to single metal nanoparticle electrocatalysts, combining two active elements by alloying in order to obtain a catalyst with an augmented activity and/or improved selectivity toward the desired product has received striking attention (Kim *et al.*, 2014 ▸).

A bimetallic compound can appear either as an alloy with a statistical distribution of its constituents over the atomic sites or as an intermetallic phase with ordered atomic sites. In the binary Au–Cu system, the alloy Au_1−*x*_Cu_*x*_ (*x* = 0.5) crystallizes with the same face-centered-cubic (f.c.c.) structure of gold or copper, whereas the intermetallic compound AuCu does not exhibit an f.c.c. structure. The chemical composition of the surface is the decisive parameter for a catalyst and ordering transformation in AuCu nanoparticles has been recently reported to have a strong influence on both the activity and selectivity during electrochemical CO_2_ reduction (Kim *et al.*, 2017 ▸).

Nanoparticles can occur in many different equilibrium shapes, such as spheres, cubes, octahedra and decahedra. In addition to single-crystalline nanoparticles, one can observe also the extensively studied multiply twinned particles (Ino, 1966 ▸). A fivefold twinned decahedral nanoparticle can be considered as an ensemble of five connected tetrahedrons and the direction along the fivefold axis is the 〈110〉 axis of the f.c.c. structure (Fig. 1 ▸). Filling the space with the multiply twinned sub-crystals creates inevitably a gap of certain size that leads to the formation of an inner-strained field. The quantitative description for f.c.c. metals is the missing angle, which can have a positive value for an existent gap or a negative value if the assembly of the five segments leads to an overlap. For the f.c.c. metals, *e.g.* Au and Cu, the missing angle is +7.35°, but for the intermetallic fivefold twinned nanoparticles, this structural characteristic is not well known. Considering the importance of bimetallic systems to create highly active nanoparticle catalysts, the correlation of activity and structural features is the key for a rational development of future nanoscopic materials.

Writing in **IUCrJ**, Liang and Yu elucidate the structural differences between single metal and intermetallic multiply twinned decahedral particles on the example of AuCu nanoparticles with an ordered structure (Liang & Yu, 2019 ▸). The results of the theoretical analysis on the microstructure indicate that body-centered tetragonal AuCu adopt a solid-angle deficiency of −13.35°, which corresponds to an overlap instead of a gap. The fundamental question for multiply twinned decahedral particles is how the gap can be filled or the overlap removed, but a general understanding of this phenomenon has not been achieved yet.

The work of Liang and Yu is an important contribution to the field and gives insight into the AuCu nanoparticle structure with the help of aberration-corrected scanning transmission electron microscopy (STEM). The authors compare their experimental findings to previously reported Au and FePt nanoparticles (Li *et al.*, 2014 ▸) and discuss the similarities and differences among AuCu and other existing nanoparticles exhibiting monometallic/intermetallic fivefold twinning. The authors could explain the mechanism of formation by analyzing both the energetic and geometric factors in connection with atomic level strain analysis. The understanding of the formation mechanism is critical for the rational development of bimetallic nanoparticles with multiply twinned structures and tailored catalytic properties. Liang and Yu present a general framework for decahedral fivefold twinned particles which opens up the opportunity to predict unknown fivefold twinned nanoparticles. This approach may be very promising in the design of future bimetallic nanoparticle catalysts.

Another shining point of the current work is the visualization of surface segregation at the atomic level by quantitative STEM analysis. The authors could prove a three-atomic layer-thick gold shell in the intermetallic multiply twinned AuCu nanoparticle with the help of aberration correction. The comparison of the simulated and experimental image showed different atomic distribution in the bimetallic nanoparticle. The detailed STEM study of Liang and Yu sheds light on the structural understanding of bimetallic nanoparticle catalysts exhibiting multiply twinned structures. This enables the accurate correlation of the catalytic properties of such nanoparticles with their structure at the atomic level.

## Figures and Tables

**Figure 1:**
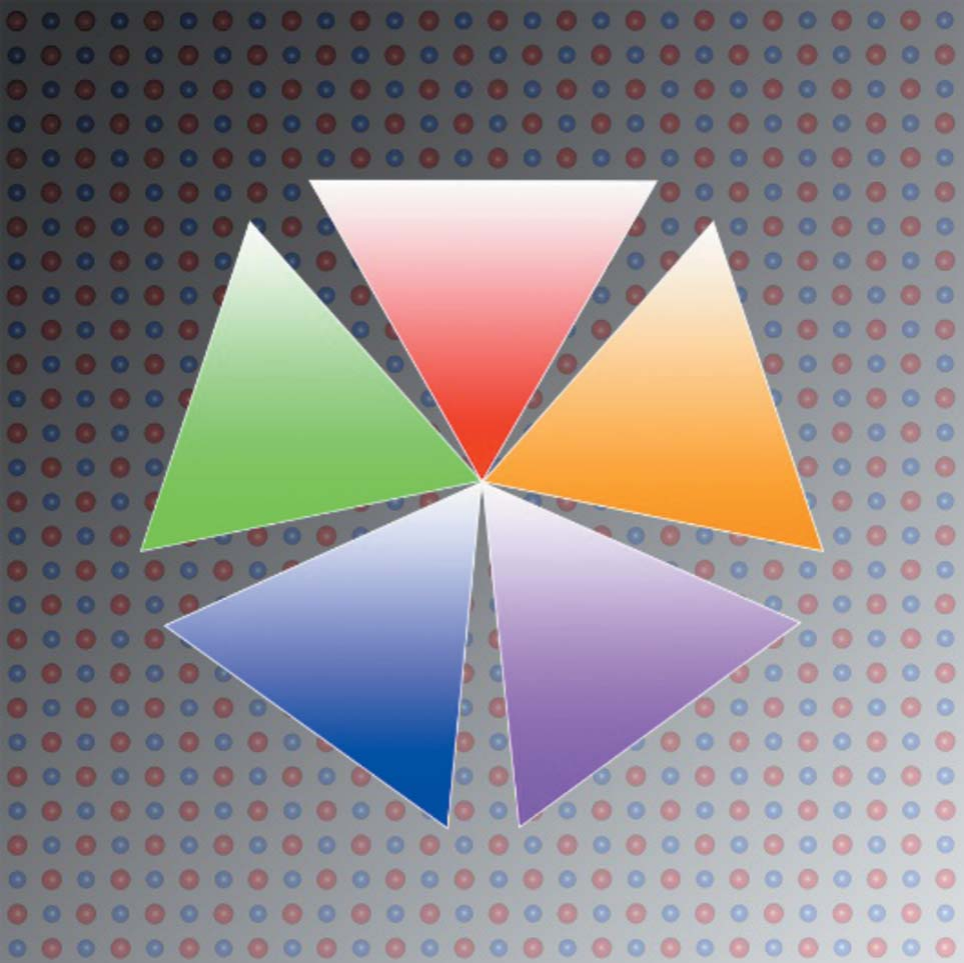
Schematic illustration of the five connected tetrahedrons.
